# Tuberculosis infection control practice and associated factors among health care workers in Ethiopia: Systematic review and meta-analysis

**DOI:** 10.1371/journal.pone.0295555

**Published:** 2023-12-12

**Authors:** Misganaw Guadie Tiruneh, Eneyew Talie Fenta, Tadele Fentabil Anagaw, Eyob Ketema Bogale, Amare Mebrat Delie

**Affiliations:** 1 Department of Health Systems and Policy, Institute of Public Health, College of Medicine and Health Sciences, University of Gondar, Gondar, Ethiopia; 2 Department of Public Health, College of Medicine and Health Sciences, Injibara University, Injibara, Ethiopia; 3 Department of Health Promotion and Behavioral Science, School of Public Health, College of Medicine and Health Sciences, Bahir Dar University, Bahir Dar, Ethiopia; The University of Georgia, UNITED STATES

## Abstract

**Background:**

The poor practice of tuberculosis infection control may increase the risk of transmission of tuberculosis in healthcare settings. Thus, this study aimed to determine the pooled magnitude of good tuberculosis infection control practice and associated factors among healthcare workers in Ethiopia.

**Methods:**

The Preferred Reporting Items for Systematic Reviews and Meta-Analysis (PRISMA) checklist guideline was followed for this review and meta-analysis. The electronic databases (Pub Med, Cochrane Library, Google scholar and grey literatures) were searched to retrieve articles by using keywords. The Joanna Briggs Institute Meta-Analysis of Statistics Assessment and Review Instrument was used to assess the quality of studies. Heterogeneity was assessed using the I^2^ statistic. The meta-analysis with a 95% confidence interval using STATA 17 software was computed to present the pooled practice and odds ratio of the determinant factors. Publication bias was assessed visually by inspecting the funnel plot asymmetry and using statistical tests using the eggers and begs test.

**Results:**

Seven studies were included in this meta-analysis, with a total of 3256 health workers. The overall pooled magnitude of good tuberculosis infection control practice was 46.44% (95% CI: 34.21%, 58.67%). In subgroup analysis, the highest practice was in Addis Ababa 51.40% (95% CI: 47.40, 55.40%) and the lowest prevalence of tuberculosis infection control practice was in Amhara region 40.24% (95% CI: 15.46, 65.02%). Working in TB clinics (AOR; 7.42, 95% CI: 3.89, 14.13) and good TB related knowledge (AOR; 4.40, 95% CI: 1.76, 10.97) were the significant predictors of good TB infection control practice.

**Conclusions:**

Only less than half of the health care workers had good practice of TB infection control. Working in TB clinics and having good TB related knowledge were statistically significant predictors of TB infection control practice. Periodic shifting of health care workers to work in TB clinics and an emphasis on TB infection control related skill based training was recommended to increase the TB infection control practice.

## Introduction

Tuberculosis (TB) is a communicable disease that is a leading cause of illness and death worldwide. Until the outbreak of the coronavirus (COVID-19), it was the leading cause of death from a single infectious agent, ranking above HIV/AIDS [1]. TB is caused by the bacillus Mycobacterium tuberculosis [1–3] which is spread from person to person through the air when people with pulmonary TB sneeze, cough, or release the TB germs into the air. A human only needs to inhale a few of these bacteria to become infected [1, 3–5].

It is estimated that approximately a quarter of the world’s population has been infected with TB bacteria, but most people will not go on to develop TB disease, and some will clear the infection. In 2021, an estimated 10.6 million people worldwide fell ill with tuberculosis (TB) [1]. A total of 1.6 million deaths occurred due to TB in 2021 (including 187,000 people with HIV) [1].

According to the global 2020 TB report, Ethiopia is among the 30 high TB and TB/HIV burden countries, with an estimated annual TB incidence of 140 per 100,000 populations and a mortality rate of 19 per 100,000 populations. Among reported TB patients, it was estimated that 1.1% of new TB patients and 7.5% of previously treated TB patients had multi-drug resistant tuberculosis (MDR-TB) in 2019 [3].

Tuberculosis infection control (TBIC) is a combination of efforts designed to reduce the risk of tuberculosis (TB) transmission within populations [4–13]. It is a subcomponent of the latest World Health Organization’s (WHO) Stop TB Strategy and helps to intensify health systems. The foundations for TBIC are early and rapid diagnosis and proper management of TB patients [11–13].

Health care facilities have been identified as highly susceptible to TB transmissions. Healthcare workers (HCWs) are not adequately protected from TB infection in health-care facilities where infection control protocols are not followed completely [14]. Health facilities are also likely to host high numbers of people with undiagnosed active TB [15], which causes a high TB transmission risk for healthcare providers, and potentially patients [16, 17]. HCWs have the potential to contact with TB patients and are the main stakeholders in health care settings to implement TB infection control [7], and the poor practice of TB infection control may increase the risk of transmission of TB in health care settings [6, 18–20].

The prevalence of TB among HCWs was (42.3% in nurses, 39.4% in doctors, and 11.3% in administrative staffs) in China [21]. A systematic review in seven high burden countries showed that the pooled prevalence of Latent Tuberculosis Infection (LTBI) was 47%, with the lowest in Brazil 37% and the highest in South Africa 64% [22]. In different parts of the world, the prevalence of TB among health professionals was higher than the general population [23–26]. A systemic review including all economic categories of 16 countries showed that the prevalence of LTBI among HCWs was 37%, the mean incidence rate of TB disease was 97/100,000 per year, and the incidence rate ratio for active TB among HCWs compared with the general population was 2.94 [15].

In Sub-Saharan Africa, it is the main concern of HCWs. The median prevalence of LTBI in HCWs was 62%, and 387/100,000 was the median incidence of TB cases in Sub-Saharan Africa [27] and the prevalence of MDR-TB among HCWs in South Africa was 13% [28]. A study from Rwanda revealed that the prevalence of LTBI was 69% among HCWs compared to 39% in school workers [26]. Additionally, a finding from Kenya also showed that the prevalence of LTBI was 60% among HCWs compared to school workers (48.2%) [29].

After the initiation of chemotherapy for drug-resistant TB, infection prevention measures for TB have been ignored by healthcare workers (HCWs) in health care settings [30], which increases the risk of nosocomial transmission of tuberculosis [2]. Different studies have shown a different level of health care workers TB infection control practice in Ethiopia, ranging from 19.6% to 72.4% [4–6, 8–10, 30–33]. To achieve the national target of 91 per 100,000 population by 2025 [3], comprehensive estimation of TB infection control practice is essential. However, there is no consistent conclusion on the TB infection prevention and control practice among health care workers in Ethiopia. Therefore, this study aimed to determine the magnitude of good TB infection control practice among health care workers in Ethiopia through a systematic review and meta-analysis, which would provide evidence of the existing occupational risk of infection in health care settings, increase awareness among HCWs to implement and practice essential infection control measures, and it will also help policymakers to adopt and implement necessary TB prevention and control measures to reduce the disease burden.

## Methods

### Search strategy and information sources

This systematic review and meta-analysis was conducted by following the Preferred Reporting Items for Systematic Reviews and Meta-Analyses (PRISMA) guidelines [34]. Electronic databases (PubMed, Cochrane Library, and Google Scholar) were used to search and retrieve related articles. The grey literatures were also searched in institutional repositories and research gate. The literature search technique was conducted by using the keywords (“tuberculosis”) AND (“infection control practice” OR “infection prevention practice” OR “disease control practice” OR “disease prevention practice”) AND (“Health care worker” OR “Health personnel” OR “Health professional”) AND (“Ethiopia” OR “Ethiopian”). No limits were applied for publication date coverage. The retrieved study references were also screened and checked manually. The protocol for this systematic review and meta-analysis was registered in the International Prospective Register of Systematic Reviews (PROSPERO) and obtained a registration number CRD42023393580.

### Eligibility criteria

For the review, CoCoPop mnemonic (Condition, Context and Population) approach was used. Based on the PICO statement: Population: Healthcare workers in Ethiopia; Intervention: Studies reporting TBIC practices in Ethiopia; Comparison: studies reporting TBIC practices outside Ethiopia; Outcome: Proportion of TBIC practice was also considered. Studies that reported TB infection control practice and associated determinants among Ethiopian health care workers using any type of study design at any health facility level, studies with open access to full text and written in English language were included. Studies done outside of Ethiopia, studies without abstract or full text, reports, and qualitative studies were excluded. Due to a lack of sufficient information for quality evaluation or data extraction, conference summaries were also removed. All studies starting from beginning to January 2023 were included. Articles were assessed for inclusion using their title and abstract, and then a full review of the articles was done before they were included in the final review.

### Data extraction and management

Eligible studies were imported to Endnote v.9 and duplicates were removed. The three independent reviewers (MG, TF and EK) were did the abstract, full text review, and data abstraction. Any disagreements were resolved by consensus and the involvement of the two reviewers (AM and ET). Full text articles were retrieved for studies that meet the inclusion criteria. Data extraction was performed by three independent reviewers using the data extraction form. The following data were extracted: author, publication year, study design, place of study, sample size, and participants.

### Quality assessment

The Joanna Briggs Institute (JBI) critical appraisal check list was used to assess the quality of studies which is freely available at https://jbi.global/critical-appraisal-tools. Using the tool as a protocol, the three reviewers (MG, ET and EK) evaluate the quality of the original articles independently. Those studies, scores 5 or more in JBI criteria were considered to have good quality and included in the review [35]. Discrepancies in the quality assessment were resolved through the involvement of the authors (TF and AM). One study was rejected due to the quality rating.

### Statistical analysis

The necessary data were extracted from the studies using Microsoft Excel V.2016, and the extracted data were exported to STATA version 17 software for analysis. The articles were summarized by tables and forest plot. The standard error and 95% confidence interva1 for the proportion of good TB infection control practice was calculated for those studies in which estimates of standard error and 95% confidence interval for the proportion of good TB infection control practice were not found in their full text of their article.

When the meta-analysis performed, the significance of the pooled Odds Ratio (OR) was determined by Z-test. Random effect model was applied to determine the pooled magnitude for each study because of significant heterogeneity exists. The statistical heterogeneity was checked subjectively by using forest plot, and objectively by Cochrane Q-test and I^2^ statistics [36]. Meta-analysis for the pooled proportion of TB infection control practice showed there was significant heterogeneity among the studies (I^2^ = 98.23%, Q = 294.2, p <0.001) as a result a random effects meta-analysis model was applied to estimate the pooled effect.

Publication bias was tested by visually inspecting a funnel plot and it was symmetric [37]. Furthermore, Egger’s test [38], showing a P-value of 0.0068 and Begg’s test [39], showing P-value 0.13, thereby indicating a low risk of publication bias. Subgroup analysis based on region and study design was also done.

## Result

**Fig 1:**
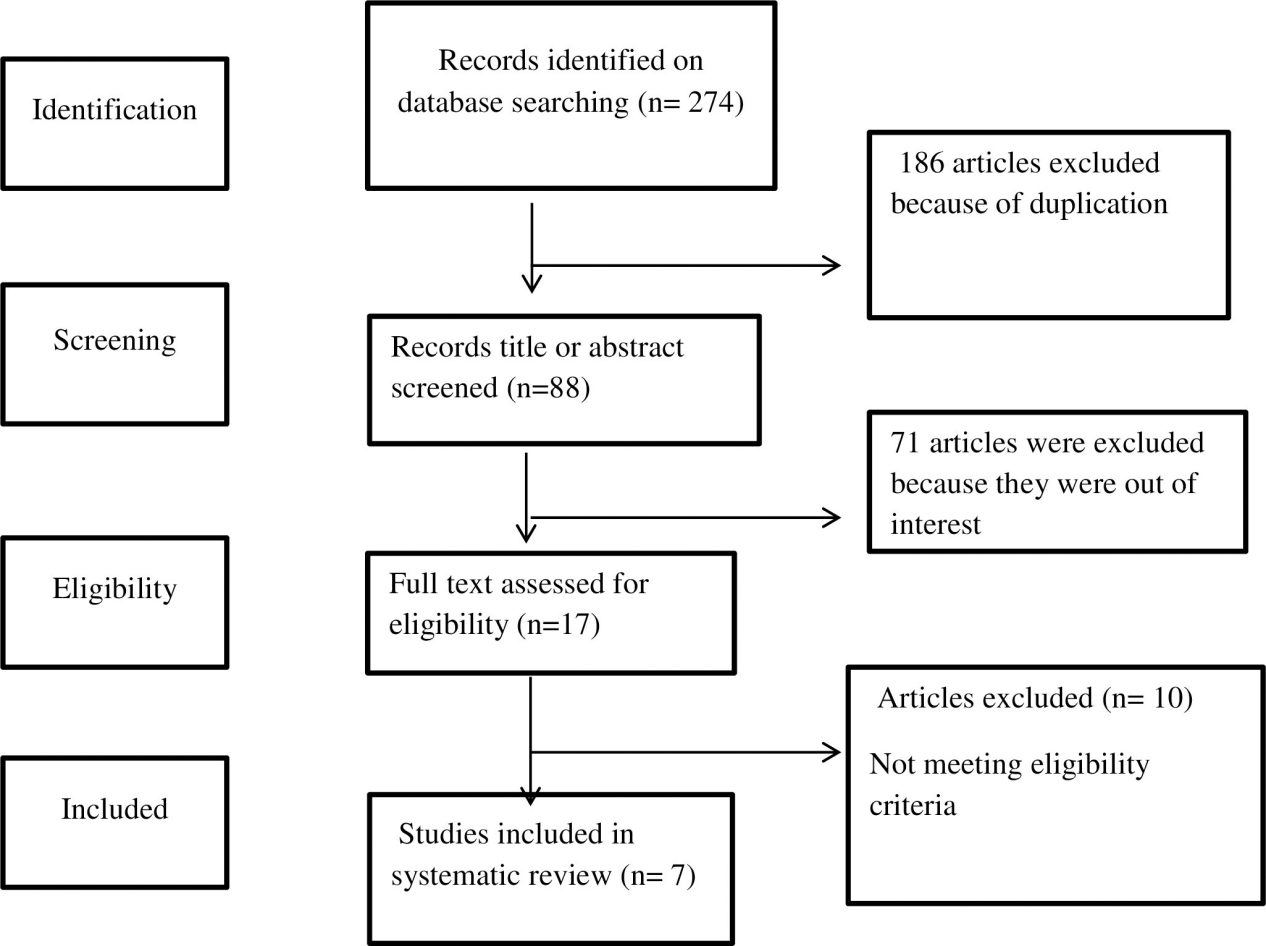
PRISMA flow diagram of study selection.

A total of 270 records were identified through our initial database search. After duplicate records were removed, 88 records were reviewed by title and abstract. Seventeen articles were included for full text review. Finally, seven studies were included in the review after applying inclusion and exclusion criteria (Fig 1). No additional studies were obtained after retrieving the references of all the 17 full text reviewed articles.

### Study characteristics

In the meta-analysis, seven studies were included. Three (42.8%) of the studies were from Amhara [5, 6, 31], three in SNNP regions [9, 10, 33], and one in Addis Ababa [4]. All of the studies were cross-sectional [4–6, 9, 10, 33], except one study which was hybrid [31]. Of all the studies, four of them were conducted in hospitals [6, 10, 31, 33], two were both in hospitals and health centers [4, 9], and one in health center [5]. The total sample size for this review was 3256 with a maximum sample size of 728 [9], and a minimum sample of 198 in SNNP Region [33] (Table 1).

**Table 1:** Characteristics of included studies in the meta-analysis of the pooled magnitude of TB infection control practice among health care workers in Ethiopia.

Authors/ publication year	Region	Study design	Study setting	Sample size	Magnitude of good TBIC practice (N)	Quality assessment based on JBI
Tamir T et al., 2016 [5]	Amhara	Mixed CS	HC	647	246	8
Tadesse AW et al., 2020 [10]	SNNP	CS	Hospitals	411	164	7
Labena f et al., 2021 [9]	SNNP	Mixed CS	HC & hospitals	728	327	8
Temesgen C & Demissie M., 2014 [31]	Amhara	Hybrid	Hospitals	313	204	6
Gizaw DG et al., 2015 [4]	Addis Ababa	CS	HC & hospitals	582	282	7
Alene KA et al., 2019 [6]	Amhara	CS	Hospitals	377	74	7
Wondimu et al., 2021 [33]	SNNP	CS	Hospitals	198	127	8

CS: Cross-sectional, HC: Health Center, JBI: Joanna Brigg’s Institute, SNNP: Southern Nation Nationalities and Peoples.

### Pooled prevalence of Tuberculosis infection control practice

**Fig 2:**
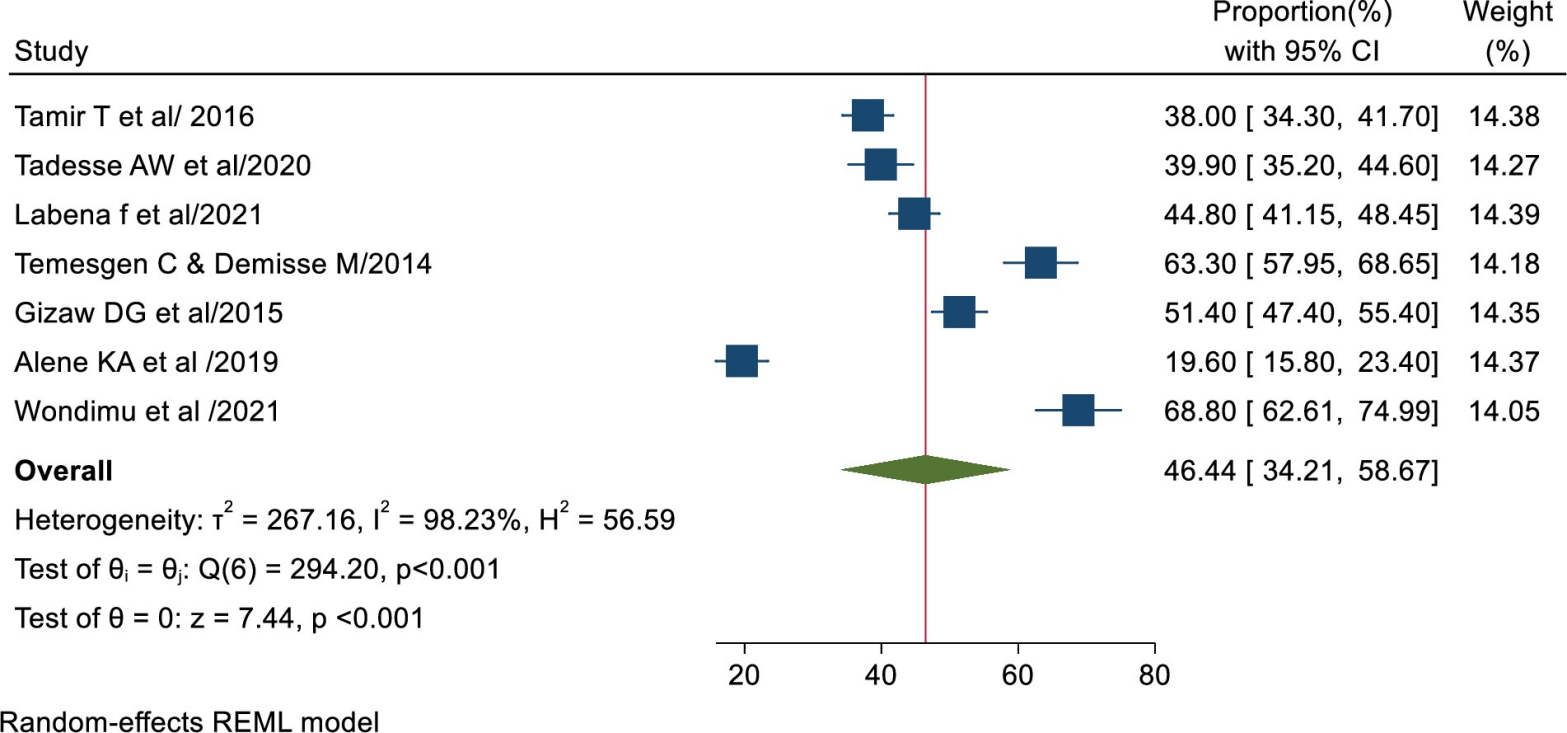
Forest plot of the pooled magnitude of tuberculosis infection control practice in Ethiopia.

The pooled magnitude of good TB infection control practice among health care workers in Ethiopia was 46.44% (95% CI 34.21%, 58.67%). Based on tau square (between studies variance), tau2 = 267.163 & I2 = 98.23 with p value < 0.00005 which indicates there is statistically significant heterogeneity among studies (Fig 2).

### Subgroup analysis based on region

**Fig 3:**
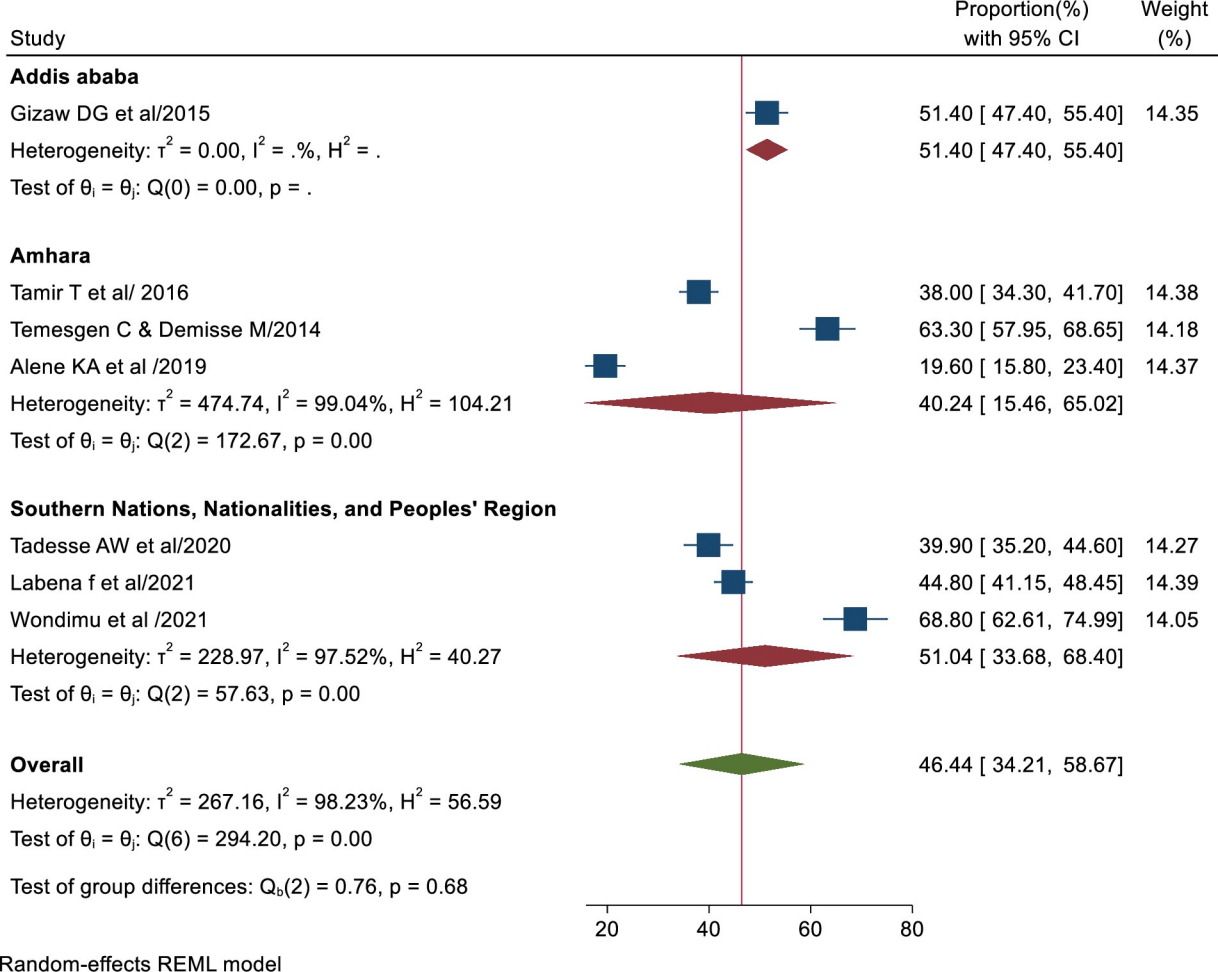
Forest plot displaying the subgroup analysis of TB infection control practice based on region.

The subgroup analysis indicated variation in TBIC practice based on region. Three of the studies were from Amhara, three in SNNPR regions, and one in Addis Ababa. In subgroup analysis, the highest magnitude of good TB infection control practice was in Addis Ababa 51.4% (95% CI: 47.40–55.40%), and the lowest magnitude of tuberculosis infection control practice was in Amhara region 40.24% (95% CI: 15.46–65.02%). In SNNP region, the magnitude of good TB infection control practice was 51.04% (95% CI: 33.68–68.40%) [4–6, 9, 10, 31, 33] (Fig 3).

### Subgroup analysis based on study design

**Fig 4:**
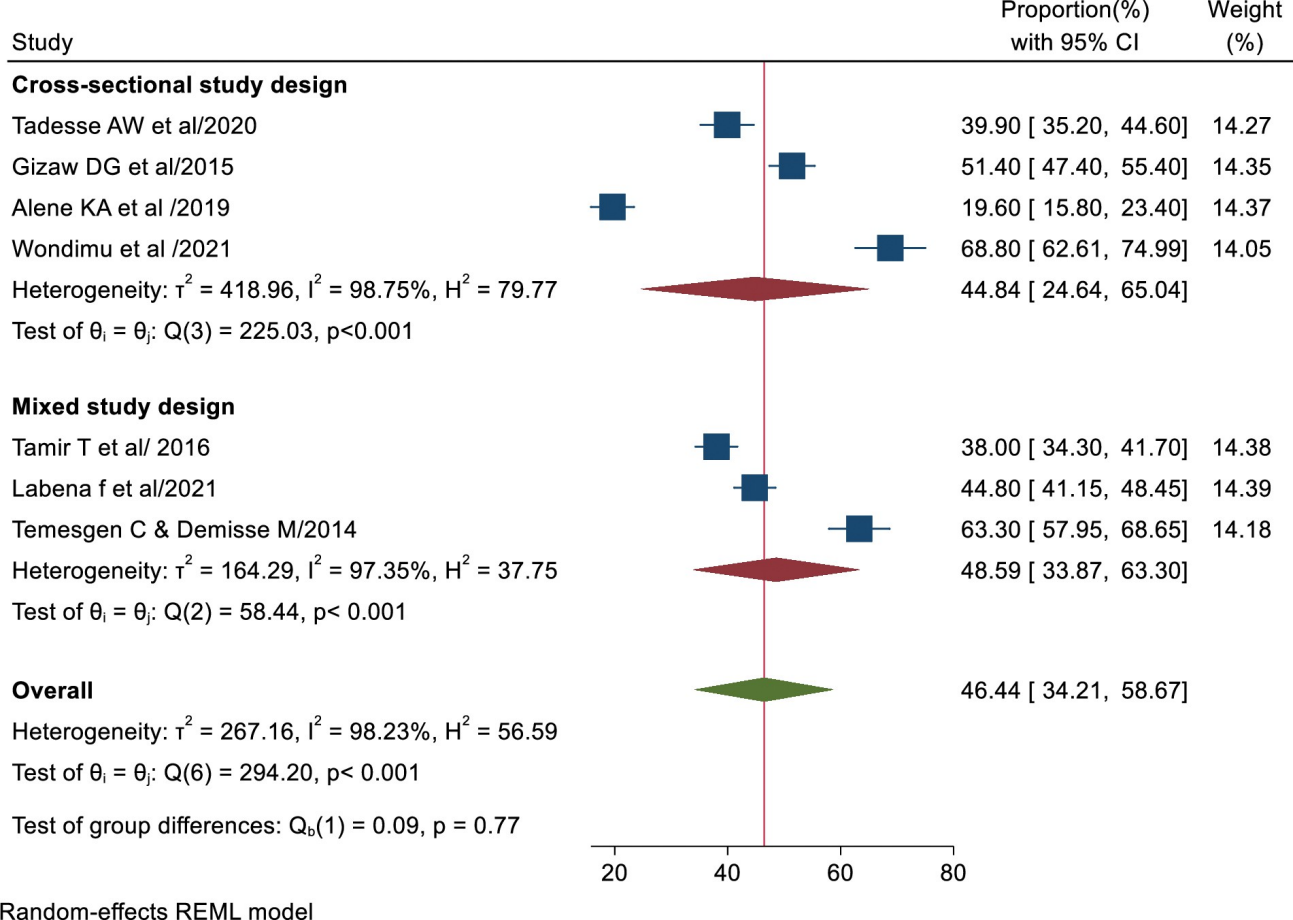
Forest plot displaying the subgroup analysis of TB infection control practice based on study design.

The subgroup analysis also revealed that there was a variation in TB infection control practice based on the study design. The pooled magnitude of TB infection control practice among health care workers in Ethiopia based on cross-sectional study design was 44.84% (95% CI: 24.64–65.04%) while using mixed study design, the pooled prevalence was 48.59% (95% CI: 33.87–63.30%) (Fig 4).

### Publication bias assessment

**Fig 5:**
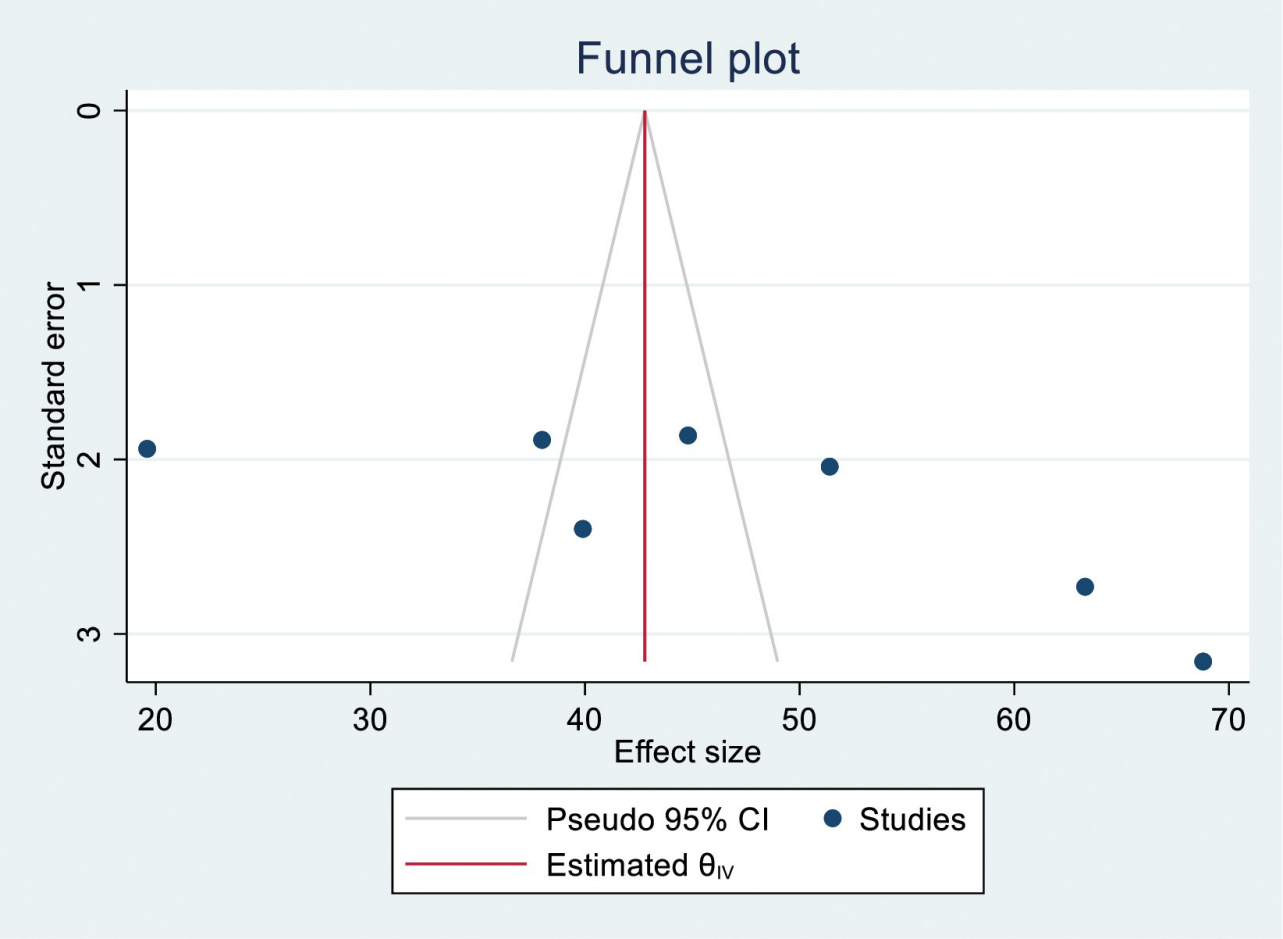
A funnel plot showing publication bias for proportion TB infection control practice.

There was no extensive asymmetry in the results of the funnel plot analysis for publication bias in the figure, as shown below (Fig 5). Furthermore, the result of non-parametric rank correlation (Begg) test for small study effects (p value = 0.133) showed no evidence of publication bias at the 0.05 level of significance. On the contrary, the regression-based Egger test for small study effects showed significant result (p value = 0.007) at 0.05 level of significance (Fig 5). Thus, publication bias could not be concluded partly because of difficulty in interpretating of the different findings from parametric and non-parametric tests.

### Sensitivity analysis

**Fig 6:**
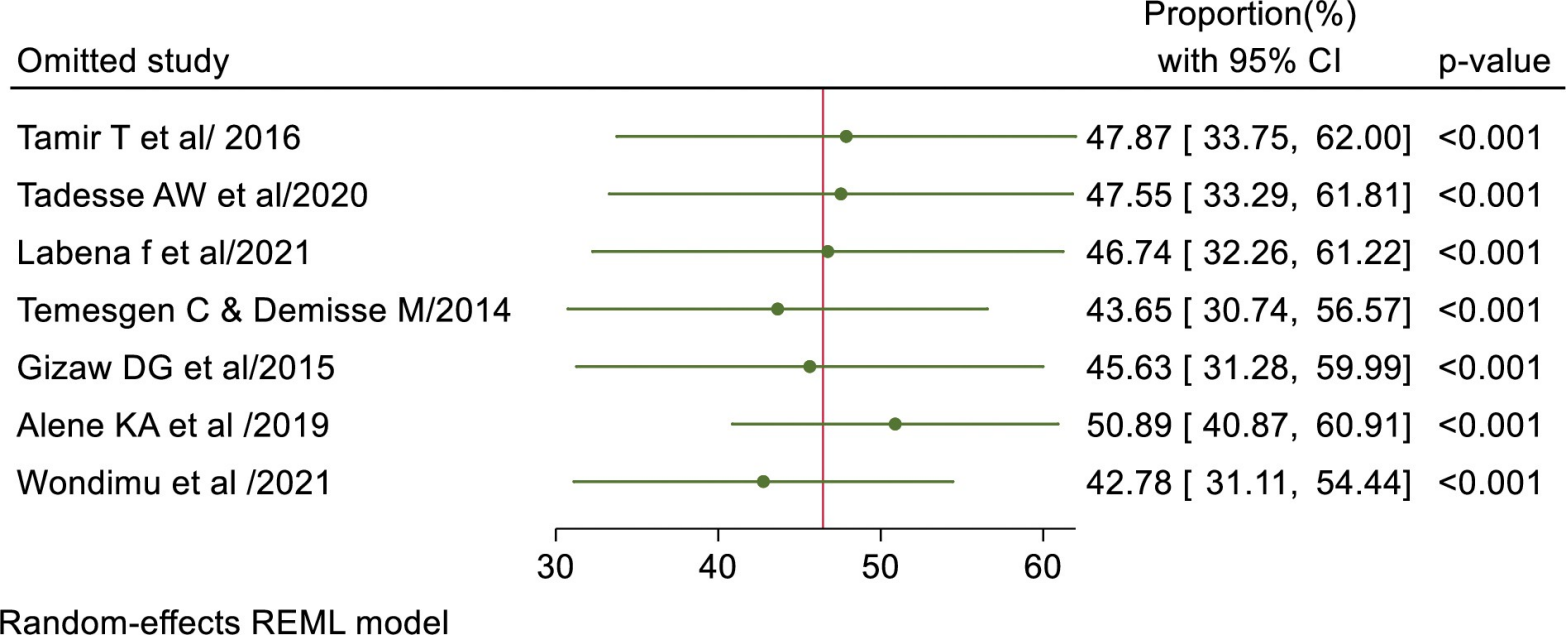
Results of leave-one-out method in sensitivity analysis for pooled proportion of TB infection control practice.

The meta leave-one-out method was conducted by eliminating each study step by step. As shown in the figure below, the pooled proportion was in the range from 42.78% (95% CI; 31.11–54.44) to 50.89% (95% CI; 40.87–60.91). The result showed that no studies were found to be outside the confidence bound of the pooled magnitude of TB infection control practice. Therefore, it showed that all studies had nearly equal influence on the overall pooled magnitude of good TBIC by excluding leave out one from meta-analysis (Fig 6).

### Factors associated with tuberculosis infection control practice

This meta-analysis assessed various factors associated with tuberculosis infection control practice in Ethiopia. Among these working in TB clinics and having good TB related knowledge were found to have a statistically significant association with good tuberculosis infection control practice. However, socio-demographic characteristics like; educational status and TB related training were not significantly associated with the outcome variable.

#### Association of working in TB clinic and tuberculosis infection control practice

**Fig 7:**
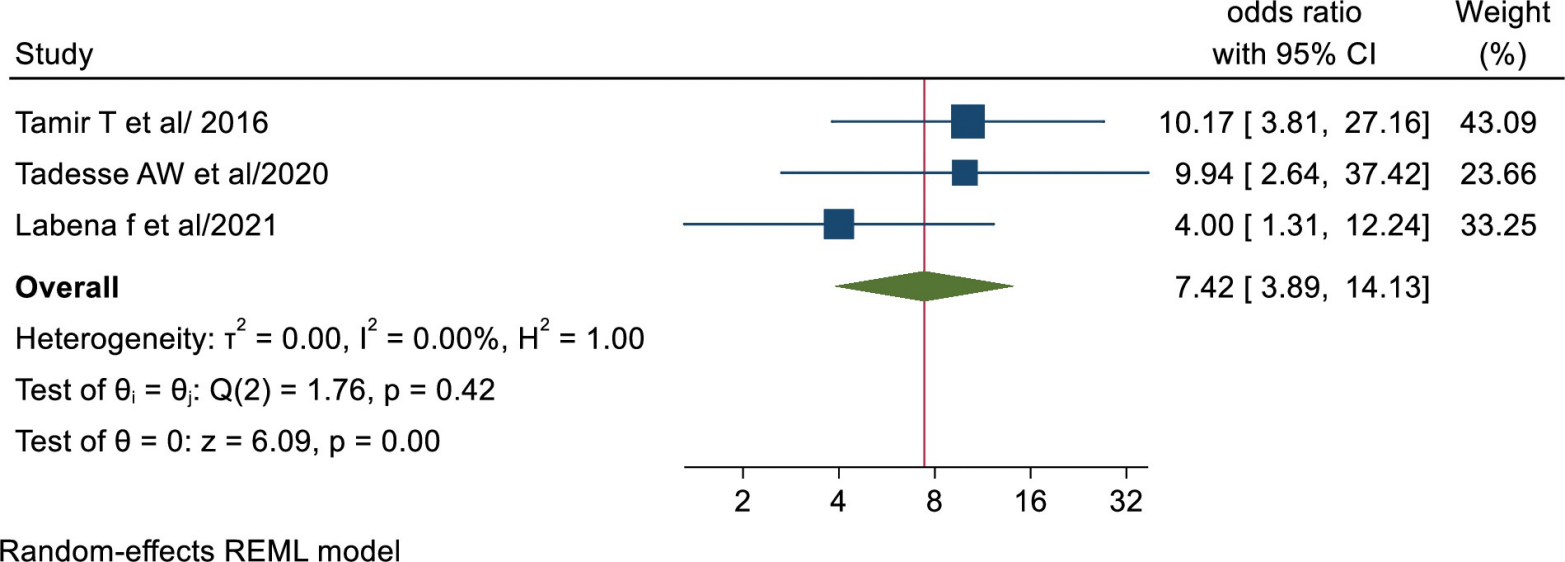
Association between health workers working in TB clinic and TB infection control practice.

In this meta-analysis, health professionals who were working in TB clinics had found a statistically significant association with TBIC practice with adjusted odds ratio (AOR; 7.42, 95% CI: 3.89–14.13). These indicated that health professionals who were working in TB clinics were 7 times more likely to have good TB infection control practice compared with their counterparts. The results of Egger’s and Begg’s tests indicated no statistically significant publication bias (P = 1.00 and P = 1.00, respectively) (Fig 7).

#### Association of TB related knowledge and tuberculosis infection control practice

**Fig 8:**
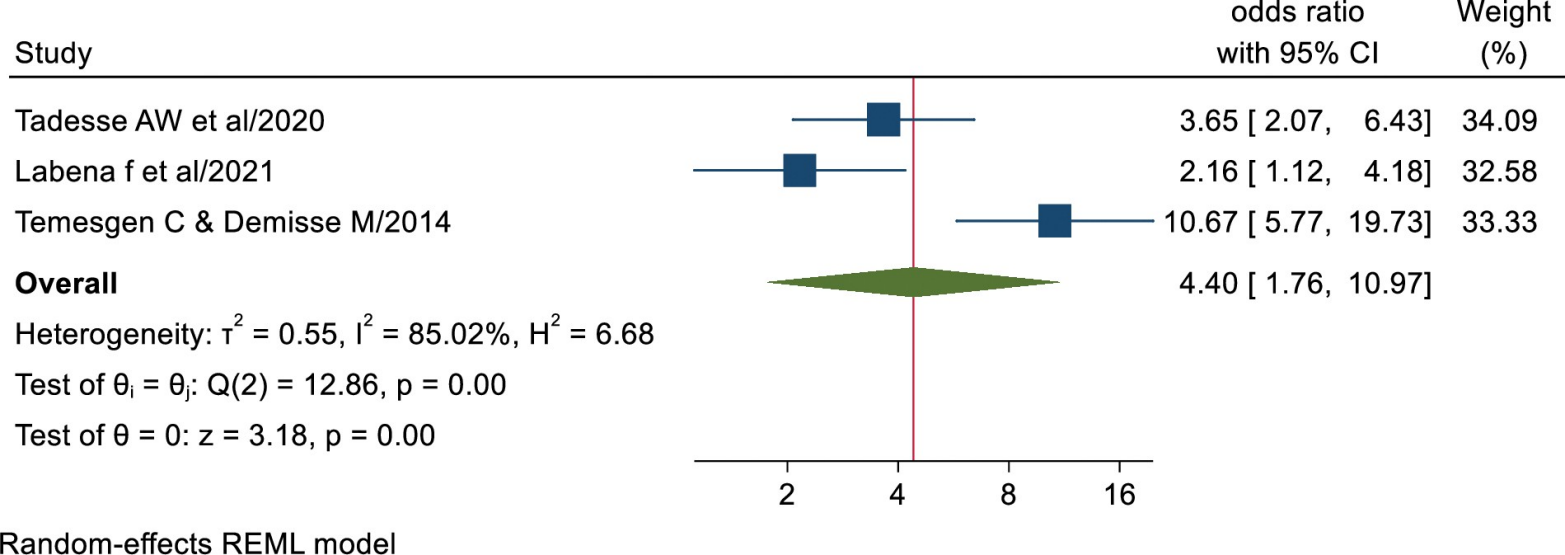
Association between health workers TB related knowledge and TB infection control practice.

Health workers who had good TB related knowledge (AOR; 4.40, 95% CI: 1.76–10.97) had statistically significant association with TB infection control practice as compared to their counterparts’. This indicates that health workers who have good TB related knowledge were 4.4 times more likely to have good TB infection control practice as compared to those who had poor TB related knowledge. The results of Egger’s and Begg’s tests indicated no statistically significant publication bias (P = 0.75 and P = 1.00, respectively) (Fig 8).

### Educational status of health professionals

**Fig 9:**
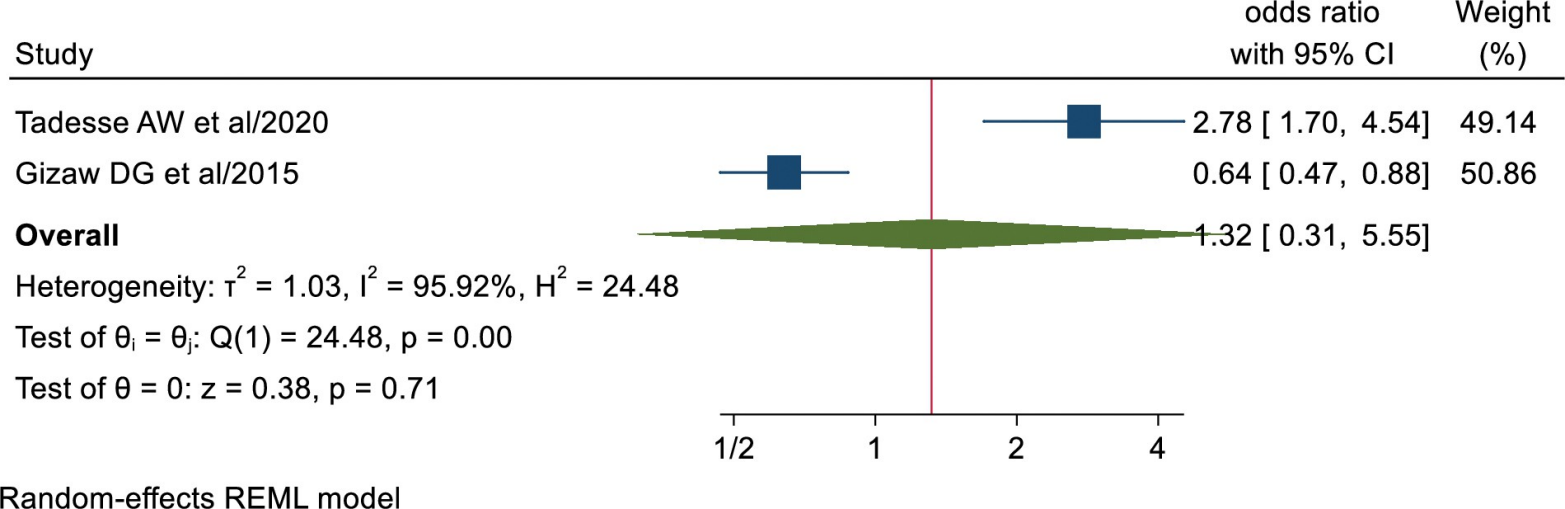
Association between health workers educational status and TB infection control practice.

According to this meta-analysis, health workers who had degree and above (AOR; 1.32, 95% CI: 0.31, 5.55) had no statistically significant association with TB infection control practice (Fig 9).

### Taking TB related training

**Fig 10:**
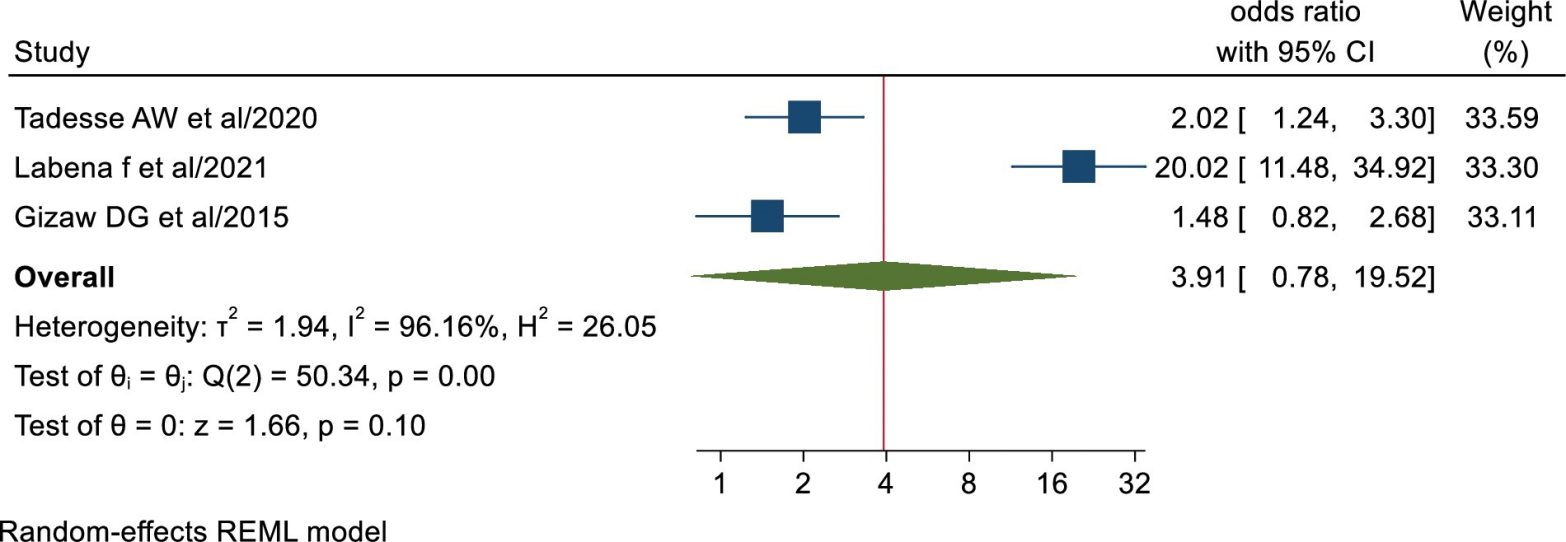
Association between health workers TB related training and TB infection control practice.

Health workers who took TB related training (AOR; 3.91, 95% CI: 0.78–19.52) were not found to have statistically significant association with TB infection control practice. The results of Egger’s and Begg’s tests indicated no statistically significant publication bias (P = 0.96 and P = 1.00, respectively) (Fig 10).

## Discussion

This systematic review with meta-analysis was performed to produce pooled estimates of the nationwide magnitude of TB infection control practices and associated factors among health care workers in Ethiopia. The focus of this review was to assess the good TB infection control practice among health care workers in Ethiopia for a better understanding of the TB control practice and to prevent the problem of TB infection throughout the country.

TB infection control measures are crucial to prevent nosocomial infection transmission risks of multidrug resistant strains to patients, and it is also important to monitor the prevalence of TB among health care workers as well as to develop prevention strategies for TB [14, 25, 32, 40]

The pooled magnitude of good TB infection control practice among health care workers in this review was 46.44% (95% CI; 34.21%, 58.67%). Individually, the magnitude of good TB infection control practice across the regions in Ethiopia ranged from 19.6% to 64.9% in the Amhara region [6, 31]. The possible explanation for the variation might be the infrastructure differences among the study settings. In this regard, health care facilities with adequate infrastructure might have good TB infection control practice [13].

The finding of this review was lower than the previous studies conducted in Nepal (59.5%) [41], South Africa (72.9%) [42], Nigeria (79.9%) [2]. The possible explanation for this variation might be attributed to our study finding was the pooled results from different studies.

The result of this meta-analysis showed that health professionals who were working in TB clinics were 7 times more likely to have good TB infection control practice compared with their counterparts. The possible explanation might be that the health care workers at primary care facilities and those working in the community may be more likely to be exposed to infectious diseases, as undiagnosed TB patients [14, 20, 21, 43, 44], which may in turn make them to adhere for good TB infection control practice. It might also be explained by due to access to TBIC related knowledge which leads to good TBIC practice.

Additionally, in this meta-analysis, health care workers who had good TB related knowledge were 4.4 times more likely to have good TB infection control practice as compared to those who had poor TB related knowledge. This finding was consistent with the previous study conducted in South Africa [42], which reported that health care workers with good TB related knowledge were positively associated with good TB infection control practice. This might be explained by developing good knowledge about TBIC measures encourages health care workers to practice the TBIC measures that enable them to protect themselves and patients from tuberculosis [10, 42]. The possible explanation for this might be that proper knowledge has been identified to be a predictor of good infection control practice and TB related training might not necessarily ensure adequate infection control practices. Rather than conventional training, skill-based training using adult learning approaches aimed at modifying HCWs’ behavior can improve TB infection control practices in health facilities [23, 41]. This might raise questions regarding the type and quality of training that was provided as it has implications for strategies to improve TB infection control practices. It is possible that the training was too theoretical rather than skill-based. It is important to not only train but also support and mentor healthcare workers on skills to strengthen the implementation of TB infection control strategies [4, 31, 42]. It implicates that improving the knowledge of health care workers about TBIC needs to be emphasized for better infection prevention and control of TB.

### Limitation

The number of studies included in this review was small, which may affected the result of the pooled magnitude of good tuberculosis infection control practices by affecting the precision.

## Conclusion

Only less than half of the health care workers had good TB infection control practice. Working in TB clinics and having good TB related knowledge were statistically significant predictors of good TB infection control practice.

Regions with low TB infection control practice should be given due attention. Further studies should be conducted to better understand the determinant factors in these regions. Shifting healthcare workers to work in TB clinics through providing skill-based training on tuberculosis infection control was also recommended.

## Abbreviations

HCWs: Health care workers
PRISMA: Preferred Reporting Items for Systematic Review and Meta-Analysis
SNNPR: Southern Nation Nationalities and People Representative
TB: Tuberculosis
TBIC: Tuberculosis Infection Control
