# Supplementary material for: Tuberculosis infection control practice and associated factors among health care workers in Ethiopia: Systematic review and meta-analysis
Source: PLoS One. 2023 Dec 12;18(12):e0295555. doi: 10.1371/journal.pone.0295555 (PMC10715661; doi:10.1371/journal.pone.0295555)
Supplement: Attachment — Submitted filename: Point by point response to Comments.pdf [file pone.0295555.s003.pdf]

**Response to editor and reviewer comments**

**Response**: The authors would like to thank the editor and reviewers for their constructive comments. The reviewer's comments improve our manuscript.

**Response for editor comments/journal requirements**

| Comments | Response | Line in revised manuscript |
|---|---|---|
| PLOS ONE style requirements | It has now been corrected as the journal style | --- |
| Overlapping text with other articles | We avoided the overlapping texts and it explained in our own way. Besides, we cited all the sources used in the manuscript and it has now been corrected. | --- |
| Data availability statement | Thank you!<br>We corrected as suggested by the journal guideline. | 359-360 |
| ORCID ID for corresponding author | We added the ORCID ID for the corresponding author. | |

**Response for Reviewer 1 comments**

**Response**: The authors would like to thank the reviewers for their constructive and valuable comments. The reviewer's comments improve our manuscript.

| Comments | Response | Line in revised manuscript |
|---|---|---|
| **Abstract**<br>1. Brief the interest of the research; is that good TBIC practice or poor TBIC practice? | Thank you! Change made as our interest was good TBIC. | Throughout the document |
| 2. What type of intervention do you recommend to enhance TBIC practice? | We accepted the comment and we recommend specific intervention | 45-46 |
| 3. Line 78-79, present the burden of TB on HCWs in comparison to general population. Is the prevalence of MDR-TB in South Africa among HCWs or the general population? | This has been specifically stated as suggested now.<br>It was among HCWs | 90-91 |
| 4. Line 80-81: your statement of problem lacks coherence. In this paragraph you started by stating the burden of TB among HCWs, and again you talked about the burden of TBs in the general population without comparison with HCWs. Therefore, modify it. | We accepted the comment and corrected as suggested. | 80-89 |
| **Methods**<br>1. Line 131-133: during data extraction, authors, place of study, study design…. were extracted. However, extraction was performed in blinded way. For what were the data extractors blind? | We accepted the comment and now it has been corrected as independent reviewers. We used "blind" to indicate there was no information sharing about the extraction process between reviewers but we accepted the comment and changed as independent. | 138-140 |
| 2. Line 136-137: how did you do blinded review approach to evaluate the quality of the original articles practically? | We used "blind" to indicate there was no information sharing about the quality assessment between reviewers but we accepted the comment and changed as independent. | 146-147 |

| | | |
|---|---|---|
| **Result**<br>1. Line 166: either cites figure-1 appropriately in the paragraph as parts of supporting information or includes it in the body of the result section and cites it accordingly. | Thank you!<br>The journal recommends inserting the caption after the paragraph in which they first cited. | |
| 2. Line 173-174: the total sample size for the review should be 3256, where do you get extra 100s? The minimum sample size is not from reference-6; recheck it. | Thank you! Change made.<br>It was happen during addition. The reference was also wrong but it has now been corrected as commented. | 184-185 |
| You miss an one important article from Oromia region which should be included according to your inclusion and exclusion criteria (View of The assessment of knowledge, attitude and practice towards tuberculosis infection control among health professionals in Nekemte referral hospital, Nekemte, Oromia, West Ethiopia: cross sectional facility based study (ijic.info) | We accepted the comment but we exclude this article due to our inclusion criteria which says “Studies that reported TB infection control practice and **associated determinants**…” but the study done in Nekemte doesn’t report the factors. | -- |
| 3. Line 175-176: either cite Table-1 appropriately in the paragraph as parts of supporting information or include it in the body of the result section and cite it accordingly. In table-1, rather than displaying proportion of good TBIC practice, it was better to state the actual number of participants with good TBIC practice for each study; therefore, anybody can compute the point pooled estimate of proportion of good TBIC practice manually. | We accepted the comment and it has now been corrected as suggested. | Table 1 |

| | | |
|---|---|---|
| 4. Line 183: similar comments on citation of figure-2. | Thank you!<br>The journal recommends appearing the title after the paragraph in which they first cited. | -- |
| 5. Line 194: similar comments on citation of figure-3. | Thank you!<br>The journal recommends appearing the title after the paragraph in which they first cited. | -- |
| 6. Line 186-201: you reported that there was significant heterogeneity among studies and you did sub-group analysis by considering region and study design as sources of variation. But you did not depict the results of your analysis in the sub-group analysis section. | It has been corrected as suggested. | 200-207<br><br>213-219 |
| 7. Line 211-2016: your way of narration for sensitivity analysis needs better way of explanation about your finding. The statement “All the results were of significance or marginal significance.” is vague. Is that to statistical significance or what? If it is to say “statistical significance”, based on what? (Given that you estimated the pooled estimate of proportion of TBIC for a single population, not difference of proportions between two population or effect sizes like OR, RR…). | We clarified it accordingly. | 232-237 |
| 8. Line 226: editorial error, “birth asphyxia” | Thank you!<br>This has now been corrected. | 247-248 |
| 9. Line230: figure-7 showed that the AOR for the association between working in TB clinic and TBIC | We extract the factors from all included studies and we pooled the odds ratio so that working in TB clinic wasn’t | |

| | | |
|---|---|---|
| practice and the pooled OR for only three studies out of seven studies. What is the reason behind? The same concern for figure-8 up to fig-10 | identified as a factor in the other four studies. | |
| **Discussion**<br>1. All over, your discussion is shallow | Thank you!<br>We tried to make stronger than before. | |
| 2. Line 266 -272: how did you come up with this justification in fact you didn't do subgroup analysis based on study setting? You have to discuss your finding with the experience of SRMA from any nation, the general population (if you retrieve), the global reports….. The same for significantly associated variables. | We accepted the comment and we tried to justify in other way. | 288-296<br>-- |
| 3. Line 273 – 278: you result and your way of explanation is no satisfactory. | We tried to add other possible justification for the association of working in TB clinic and TBIC | 301-303 |

**Response for Reviewer 2 comments**

**Response**: The authors would like to thank the reviewers for their constructive and valuable comments. The reviewer's comments improve our manuscript.

| Comments | Response | Line in revised manuscript |
|---|---|---|
| The authors did one of the most public health important issues. However, the manuscript needs revision to be considered for publication. | Thank you!<br>Really, the reviewer's comments improve our manuscript. | |
| **Minor**<br>Better to put acronyms in their full form during their first time introduction in the document. E.g LTBI | This has now been corrected as suggested. | Throughout the manuscript |
| Editorial and grammatical errors need revision. | Thank you!<br>This has now been corrected as suggested. | Throughout the manuscript |
| It's important to indicate the geographical location for the TB related magnitudes provided in the line numbers 87-89. | This systematic review was done in 16 countries without geographic restriction by including all economic class countries (lower, middle and higher income) countries. | 84-85 |
| Better to move the justification provided in line #190-192 to the discussion section. Similarly, the interpretations of odds ratio batter fit to discussion part. | Thank you!<br>This has now been corrected as suggested. | -- |
| Please use the term "good TBIC practice" uniformly throughout the document. Otherwise, when you are using only "TBIC practice" in some section it can refer to both poor and good TBIC practices, in contrary to the review's objective and overall interpretation. | Thank you! Change made.<br>This has now been corrected as suggested. | Throughout the manuscript |
| Line #174, the sample size of the cited study was not 215, rather 198. | Thank you! Change made. | 185 |
| **Major**<br>Did the individual studies | Actually two of the articles was measured the practice by | |

| | | |
|---|---|---|
| measure the practice status in a comparable way? | the mean score of items and the remaining was measured by the proportion of the number of items. However, final report was proportion. | |
| Since this is not purely prevalence or incidence studies' review, the CoCoPop criteria may not be sufficient to assess eligibility. | We tried to adjust it accordingly. | 125-127 |
| Why only the references of the included studies (seven) were assessed for eligibility? i.e Why not that of 17 potentially eligible studies? | Actually we checked the references of all full text reviewed studies and no additional study were obtained. | 175-176 |
| Any justification for the disagreement figures (TBIC range) provided in line #90-91 and table 1? | The difference is due to one of the study which is not included in the final review because of low quality. | 150-151 |
| How the final decision regarding publication bias was reached as there were contradicting results from nonparametric, parametric and forest plot based analyses? Line #203-207 | Usually, funnel plots might be interpreted differently by different observers. Publication bias couldn't be interpreted partially because of different findings from these different types of analyses and we couldn't perform meta regressions as it isn't recommended for small number of studies so that we prefer just to report the finding as some studies. However, publication bias assessment for all the identified factors was not significant. | 226-227 |
| Line #225 - 227, What do the authors mean when they say "In this meta-analysis, health professionals who were working in TB clinic had found a statistically significant association with birth asphyxia with adjusted odds ratio (AOR; 7.42, 95% CI: 3.89–14.13)." Is this editorial problem or any unexplained | Thank you!<br>This was an editorial error but it has now been corrected. | 247-248 |

| | | |
|---|---|---|
| outcome, or any other justification? | | |
| More appropriate or additional explanation for the finding given @line #273-275, could be the access to TB related training of those working in TB clinic which in turn may lead to good TBIC knowledge and practice. | Thank you!<br>Change has been made as suggested. | 302-303 |
| Recommendation provided in line #302-304 can’t be generated from this review. Because, none of the study assessed the capacity gap of HCWs. It may be due to scarcity of resource, low admin commitments, …... and others. Thus, put the strong implications in line with your findings. | Thank you!<br>Change has been made based on our findings. | 334-335 |

**Response for Reviewer 3 comments**

**Response**: The authors would like to thank the reviewers for their constructive and valuable comments.
