# Supplementary material for: Tuberculosis infection control practice and associated factors among health care workers in Ethiopia: Systematic review and meta-analysis
Source: PLoS One. 2023 Dec 12;18(12):e0295555. doi: 10.1371/journal.pone.0295555 (PMC10715661; doi:10.1371/journal.pone.0295555)
Supplement: Attachment — Submitted filename: Comments.docx [file pone.0295555.s001.docx]

Comments

Abstract

1. Brief the interest of the research; is that good TBIC practice or poor TBIC practice?
2. What type of intervention do you recommend to enhance TBIC practice?
3. Line 78-79, present the burden of TB on HCWs in comparison to general population. Is the prevalence of MDR-TB in South Africa among HCWs or the general population?
4. Line 80-81: your statement of problem lacks coherence. In this paragraph you started by stating the burden of TB among HCWs, and again you talked about the burden of HCWs in the general population without comparison with HCWs. Therefore, modify it.

Methods

1. Line 131-133: during data extraction, authors, place of study, study design…. were extracted. However, extraction was performed in blinded way. For what were the data extractors blind?
2. Line 136-137: how did you do blinded review approach to evaluate the quality of the original articles practically?

Result

1. Line 166: either cite figure-1 appropriately in the paragraph as parts of supporting information or include it in the body of the result section and cite it accordingly.
2. Line 173-174: the total sample size for the review should be 3256, where do you get extra 100s? The minimum sample size is not from reference-6; recheck it. You miss an one important article from Oromia region which should be included according to your inclusion and exclusion criteria ([View of The assessment of knowledge, attitude and practice towards tuberculosis infection control among health professionals in Nekemte referral hospital, Nekemte, Oromia, West Ethiopia: cross sectional facility based study (ijic.info)](https://ijic.info/article/view/20305/13853)
3. Line 175-176: either cite Table-1 appropriately in the paragraph as parts of supporting information or include it in the body of the result section and cite it accordingly. In table-1, rather than displaying proportion of good TBIC practice, it was better to state the actual number of participants with good TBIC practice for each study; therefore, anybody can compute the point pooled estimate of proportion of good TBIC practice manually.
4. Line 183: similar comments on citation of figure-2.
5. Line 194: similar comments on citation of figure-3.
6. Line 186-201: you reported that there was significant heterogeneity among studies and you did sub-group analysis by considering region and study design as sources of variation. But you did not depict the results of your analysis in the sub-group analysis section.
7. Line 211-2016: your way of narration for sensitivity analysis needs better way of explanation about your finding. The statement “All the results were of significance or marginal significance.” is vague. Is that to statistical significance or what? If it is to say “statistical significance”, based on what? (Given that you estimated the pooled estimate of proportion of TBIC for a single population, not difference of proportions between two population or effect sizes like OR, RR…).
8. Line 226: editorial error, “birth asphyxia”
9. Line230: figure-7 showed that the AOR for the association between working in TB clinic and TBIC practice and the pooled OR for only three studies out of seven studies. What is the reason behind? The same concern for figure-8 up to fig-10.

Discussion

1. Allover, your discussion is shallow
2. Line 266 -272: how did you come up with this justification in fact you didn’t do subgroup analysis based on study setting? You have to discuss your finding with the experience of SRMA from any nation, the general population (if you retrieve), the global reports….. The same for significantly associated variables.
3. Line 273 – 278: you result and your way of explanation is no satisfactory.
