# Supplementary material for: Tuberculosis infection control practice and associated factors among health care workers in Ethiopia: Systematic review and meta-analysis
Source: PLoS One. 2023 Dec 12;18(12):e0295555. doi: 10.1371/journal.pone.0295555 (PMC10715661; doi:10.1371/journal.pone.0295555)
Supplement: Attachment — Submitted filename: Comments.docx [file pone.0295555.s002.docx]

**Title** - Tuberculosis infection control practice and associated factors among health care

workers in Ethiopia: Systematic review and meta-analysis

**Manuscript Number** - PONE-D-23-21840

**Comments to Authors**

The authors did one of the most public health important issue. However, the manuscript needs revision to be considered for publication.

**Minor**

Better to put acronyms in their full form during their first time introduction in the document. E.g LTBI

Editorial and grammatical errors need revision.

It’s important to indicate the geographical location for the TB related magnitudes provided in the line numbers 87-89.

Better to move the justification provided in line #190-192 to the discussion section. Similarly, the interpretations of odds ratio batter fit to discussion part.

Please use the term “good TBIC practice” uniformly throughout the document. Otherwise, when you are using only “TBIC practice” in some section it can refer to both poor and good TBIC practices, in contrary to the review’s objective and overall interpretation.

Line #174, the sample size of the cited study was not 215, rather 198.

**Major**

Did the individual studies measure the practice status in a comparable way?

Since this is not purely prevalence or incidence studies’ review, the CoCoPop criteria may not be sufficient to assess eligibility.

Why only the references of the included studies (seven) were assessed for eligibility? i.e Why not that of 17 potentially eligible studies?

Any justification for the disagreement figures (TBIC range) provided in line #90-91 and table 1?

How the final decision regarding publication bias was reached as there were contradicting results from nonparametric, parametric and forest plot based analyses? Line #203-207

Line #225 - 227, What do the authors mean when they say “In this meta-analysis, health professionals who were working in TB clinic had found a statistically significant association with birth asphyxia with adjusted odds ratio (AOR; 7.42, 95% CI: 3.89–14.13).” Is this editorial problem or any unexplained outcome, or any other justification?

More appropriate or additional explanation for the finding given @line #273-275, could be the access to TB related training of those working in TB clinic which in turn may lead to good TBIC knowledge and practice.

Recommendation provided in line #302-304 can’t be generated from this review. Because, none of the study assessed the capacity gap of HCWs. It may be due to scarcity of resource, low admin commitments, …... and others. Thus, put the strong implications in line with your findings.
